# Berlin Notes

**Published:** 1874-09-01

**Authors:** M. P. Hatfield


### BERLIN NOTES.—NO. II.

### A CLINIC WITH BARON VON LANGENBECK.

*By M. P. Hatfield, M.D.*

SCENE I., 1:45 P. M.—Large, shabby amphitheatre; seats broad, wooden stairs, uncomfortable enough to have been chosen for Patience's smiling place; students scrambling for the best places; air full of tobacco smoke and expletives.

*Scene II.*, 2 P. M.—Sudden silence and respectful rising on the part of the students. Looking down in the cock-pit, we see in the midst of his attendant "practical physicians," a gray-haired, well-preserved, soldierly old man. It is Herr Prof. von Langenbeck, elegant in dress and address, and of manners most courtly—except when sorely tried; *e. g.*, he bows to the students and selects from the list one who is expected to make a diagnosis and prescribe the treatment necessary for a little baby that has just been laid upon the operating table. Herr B. has, unluckily, not made a specialty of spina bifida. He utterly fails in diagnosis, and, when, hard pressed for treatment, he suggests that a section be taken out of the spinal column, the baron's righteous indignation knows no bounds. Baby has a carbolized dressing applied, and poor B. flies incontinently to the upper back seats.

*Case No. II* is brought in upon a stretcher, and proves to be a young woman with a hideous protrusion of the left cheek. Examination reveals a tumor—probably malignant—in the antrum; hence excision of the upper jaw is determined upon. A la Nussbaum, Langenbeck then proceeds to perform tracheotomy, making fast to the tracheal tube about three feet of rubber tubing. This communicates with a chloroform inhaler, which is placed outside the crowd about the table, thus giving the one administering the anæsthesia plenty of elbow-room. The patient's mouth is now plugged; Langenbeck makes a curved incision downward from the inner angle of the eye to the tip of the ear, and removes the superior maxilla at his leisure. The hæmorrhage, of course, is great, until checked by means of hot irons, under which the tissues siss and hiss like St. Lawrence on his gridiron, but the operation was *wunderschon.* What is left of the patient's face is sewed together, a flap is brought down from her forehead to fill a gap near the inner canthus and the girl is carried away, happily unconscious of all that has happened.

[N. B.—During the whole of this operation, as in almost all that we saw in Berlin, chloroform was given without stint and seemingly pushed to a dangerous extent. Nevertheless, this woman made a good recovery, was not greatly disfigured, and at last accounts was walking about the hospital wards.]

*Case III.* is necrosis of the ankle, requiring Syme's amputation of the foot. This is performed exactly as laid down in the books, except that the schlauch-tourniquet is used. This hose tourniquet, as you may know, consists essentially of two to three feet of small rubber hose—about an inch in diameter—and a long, strong, elastic bandage. The latter, beginning at the toes, was applied so closely that nearly all the blood was driven before it out of the limb. The bit of hose was then twisted around the leg—over the femoral artery—as tightly as two men could pull it, and secured by means of a hook and chain in its ends. On removing the bandage the limb was found pale and exsanguineous, and hence the operation was almost literally bloodless. No doubt this was in part due to Langenbeck's skillful fingers, but we doubt if with any other tourniquet even the best of surgeons could have amputated a foot with so little hæmorrhage. Except a little cutaneous oozing, the cutting was as clean and easy as if it had been done upon smoked beef, and the amputation performed with a neatness and dispatch very unlike the previous operation. Then, of course, it would have been injudicious to wind a schlauch about the patient's throat, but in all operations upon the limbs the hose tourniquet has proved a valuable and efficient aid to the surgeon. But is there no drawback to its use? Yes, there is always somewhere a weakest spot, and here it consists in possible paralysis. We have no account of any bad effects following its use in amputations, but on looking over our notes we find a case where paralysis seemed to result from its use in a tedious operation for anchylosis of the elbow. In lecturing upon this case Langenbeck alluded to another, in private practice, where persistent partial paralysis of the hand occurred after prolonged pressure of the rubber tube upon the brachial plexus. These were the only cases in which he had observed evil results, and with these two exceptions—both in the upper extremity—Herr Prof. von Langenbeck has always secured the most fortunate results, and esteems the schlauch tourniquet as one of the most valuable discoveries of modern surgery.

